# Lipoarabinomannan in urine during tuberculosis treatment: association with host and pathogen factors and mycobacteriuria

**DOI:** 10.1186/1471-2334-12-47

**Published:** 2012-02-27

**Authors:** Robin Wood, Kimberly Racow, Linda-Gail Bekker, Keren Middelkoop, Monica Vogt, Barry N Kreiswirth, Stephen D Lawn

**Affiliations:** 1 Desmond Tutu HIV Centre, Institute of Infectious Diseases and Molecular Medicine, University of Cape Town Faculty of Health Sciences, Cape Town, South Africa; 2 Department of Medicine, University of Cape Town Faculty of Health Sciences, Cape Town, South Africa; 3 Department of Science and Technology/National Research, Foundation Centre of Excellence in Epidemiological Modelling and Analysis, University of Stellenbosch, Stellenbosch, South Africa; 4 Public Health Research Institute TB Center, University of Medicine and Dentistry of New Jersey, Newark, NJ, USA; 5 Department of Clinical Research, Faculty of Infectious and Tropical Diseases, London School of Hygiene and Tropical Medicine, London, UK

## Abstract

**Background:**

Detection of lipoarabinomannan (LAM), a *Mycobacterium tuberculosis *(*Mtb*) cell wall antigen, is a potentially attractive diagnostic. However, the LAM-ELISA assay has demonstrated variable sensitivity in diagnosing TB in diverse clinical populations. We therefore explored pathogen and host factors potentially impacting LAM detection.

**Methods:**

LAM-ELISA assay testing, sputum smear and culture status, HIV status, CD4 cell count, proteinuria and TB outcomes were prospectively determined in adults diagnosed with TB and commencing TB treatment at a South African township TB clinic. Sputum TB isolates were characterised by IS*61110*-based restriction fragment length polymorphism (RFLP) and urines were tested for mycobacteriuria by Xpert^® ^MTB/RIF assay.

**Results:**

32/199 (16.1%) of patients tested LAM-ELISA positive. Median optical density and proportion testing LAM positive remained unchanged during 2 weeks of treatment and then declined over 24 weeks. LAM was associated with positive sputum smear and culture status, HIV infection and low CD4 cell counts but not proteinuria, RFLP strain or TB treatment outcome. The sensitivity of LAM for TB in HIV-infected patients with CD4 counts of ≥ 200, 100-199, 50-99, and < 50 cells/μl, was 15.2%, 32%, 42.9%, and 69.2% respectively. Mycobacteriuria was found in 15/32 (46.9%) of LAM positive patients and in none of the LAM negative controls.

**Conclusions:**

Urinary LAM was related to host immune factors, was unrelated to *Mtb *strain and declined steadily after an initial 2 weeks of TB treatment. The strong association of urine LAM with mycobacteriuria is a new finding, indicating frequent TB involvement of the renal tract in advanced HIV infection.

## Background

Lipoarabinomannan (LAM), a major lipopolysaccharide component of the cell wall of the genus *Mycobacterium *and related *actinomyces*, was first characterised in the 1980's [1]. LAM is present at the cell surface where it can readily interact with host receptors and act as an immunomodulator [2,3]. LAM is also highly immunogenic and anti-LAM antibodies are produced during mycobacterial infection [4]. The detection of anti-LAM antibodies has been proposed for diagnosis of active tuberculosis [5,6]. Both LAM antigen and anti-LAM antibody may be found aggregated in circulating antibody-antigen immune complexes [6,7].

LAM antigen is a 19,000 (± 8,500) daltons sized lipopolysaccharide which can be recovered in large quantities from *Mycobacterium tuberculosis *(*Mtb*) cultures [1], and is detectable in serum [8], sputum [9,10] and urine in a wide variety of tuberculosis (TB) clinical settings [10-19]. Urine LAM testing has shown markedly variable diagnostic accuracy for TB in field studies with a generally low sensitivity [20,21]. However, sensitivity of the assay has been reported to be increased in HIV-TB co-infected patients with advanced immune suppression [17,19] and also in those with high TB bacillary burden [17,18]. A simple, low-cost, point-of care version of this assay has been shown to have considerable utility when screening for TB prior to antiretroviral therapy [22]. In addition to host factors which may affect LAM detection such as immune status, the quantitative expression of LAM on the mycobacterial surface has also been shown to be strain dependent [23,24].

It has been postulated that LAM is released from metabolically active or degrading mycobacterial organisms into the serum, with subsequent filtration by the kidneys, passing into the urine where it can be detected by enzyme-linked immunosorbent assay (ELISA) [14]. The molecular size of LAM is similar to myoglobin (16,700 daltons), the primary oxygen carrying hemoprotein in striated muscle [25], which readily passes through the normal glomerulus into urine following muscle injury [25]. However, in contrast to myoglobin, LAM is a highly immunogenic molecule frequently associated with anti-LAM antibodies readily detectable in serum [4-6]. Systemically released LAM may therefore circulate in large immune complexes [26], which would not be able to pass through normal renal glomeruli to the urine [27]. In contrast, free or antibody-complexed LAM released from mycobacteria within the renal tract could pass directly into urine without the need to pass though the glomerular membrane.

We therefore hypothesised that the variable sensitivity of the urine LAM assay for TB diagnosis may be determined by a variety of pathogen and host factors. We first explored temporal changes in urine LAM during TB therapy when increased mycobacterial cell killing [28] would be expected to increase LAM release. We then explored the relationship between urine LAM and host factors, including HIV status, level of immune suppression, renal proteinuria and response to treatment. To explore relationships between LAM and mycobacterial factors we compared sputum TB strain patterns and the presence of mycobacteriuria in LAM positive patients and LAM negative patients.

## Methods

### Study site and population

The study population consisted of sequential adult TB patients presenting between September 2009 and April 2011 to the TB clinic of a peri-urban township which has been described elsewhere [29]. All patients were diagnosed with TB, notified to the South African National TB Control Programme (NTBCP) and commenced on standard rifampicin based short course TB treatment administered under directly observed supervision [30]. Routine TB diagnostic and treatment procedures were performed as outlined by the NTBCP [28]. Additional TB and HIV information was collected prospectively in case report forms and by review of the local TB and HIV registers. Written informed consent was obtained from all patients and the study was approved by the Research Ethics Committee of the Faculty of Health Sciences of the University of Cape Town. No previously reported studies have examined the kinetics of urine LAM during TB treatment. We chose a convenience sample size of 200 which was based primarily on the average number of TB patients presenting at Masiphumelele Clinic per year and also to ensure a diverse study population in terms of sputum and HIV status.

### Sample collection and storage

Patients were requested to provide a urine sample at each routine visit to the TB clinic every day during week 1 and on week 2, week 8, week 16, and week 24 of TB treatment. Urine samples were provided in separate sterile containers, aliqotted into 5-7 ml containers, and refrigerated at -20^0 ^C for later analysis for LAM-ELISA, urinary protein:creatinine ratio (P:C ratios) and Xpert^® ^MTB/RIF [31]. The aforementioned analyses were performed in accredited laboratories of the South African National Health Laboratory Service (NHLS). Sputum samples collected for direct sputum smears during the course of routine management were additionally requested to be cultured for *Mtb *at an accredited TB laboratory of the NHLS.

### Urinary LAM ELISA analysis

LAM ELISA testing was performed strictly according to manufacturer's instructions (Clearview^® ^TB ELISA, Alere Health Services, USA). Assays were performed in duplicate together with negative controls. Samples were considered positive if the mean optical density (OD) value minus the control OD value was greater than or equal to 0.1 and negative if the mean OD value minus the control OD value was less than 0.1. Patients were categorised as LAM positive if their initial pre-treatment (day 1) urine sample was positive as per conventional screening procedure [10-19]. During the study we collected an additional 10 urine samples at specific time points throughout TB treatment, which increased the probability of acquiring false positive results. We therefore adjusted our case definition to include patients who tested LAM positive at day 1 and those who tested LAM negative at day 1 but subsequently tested LAM positive on two or more occasions during TB treatment.

### Urinary protein and creatinine analysis

The P:C ratio (units = g/mmol) on a single urine specimen provides an estimate of approximate daily total protein excretion [32]. LAM positive and an equal number of LAM negative control urines were analysed for protein (g) and creatinine (mmol) to estimate P:C ratios on day 1 and week 24 specimens. LAM negative controls were chosen from HIV positive patients with laboratory confirmed TB using a random number table. If day 1 urine was not available, the earliest available specimen from the first week was used for analysis. Missing week 24 urine specimens were not replaced.

### Restriction fragment length polymorphism (RFLP) analysis

Pre-treatment sputum isolates that tested positive for *Mtb *by culture were inoculated in duplicate into 7H9 liquid medium supplemented with oleic acid, albumin, dextrose, and catalase (OADC) and 15% glycerol and then stored at -70°C. Frozen duplicate culture stock was shipped to the Public Health Research Institute (PHRI) Tuberculosis Center at University of Medicine and Dentistry of New Jersey (UMDNJ). Culture stocks were sub-cultured on Lowenstein-Jensen slants, and DNA was extracted from each isolate. IS*6110*-based RFLP analysis was performed as described elsewhere [33]. RFLP patterns were analyzed using Bio Image pattern matching software (BioImage Systems Inc., Jackson, Michigan, USA.). IS*6110-*based RFLP derived DNA fingerprints were assigned a strain code following a nomenclature system that has been described elsewhere [34]. TB RFLP strain patterns from LAM positive and LAM negative patients were compared with each other and with previously described strains from this community [35,36].

### Urine Xpert^® ^MTB/RIF analysis

Single pre-treatment aliquots of urine from patients who tested LAM positive and an equal number of randomly matched LAM negative controls, as described above, were analysed with the Xpert^® ^MTB/RIF assay. In order to further increase the yield of the Xpert^® ^MTB/RIF assay for patients testing LAM positive but Xpert^® ^MTB/RIF negative, urine collected from LAM positive, Xpert negative patients during the first week of TB treatment were pooled and re-tested. Each patient provided up to 5 daily urine samples of 4 ml each, which were thawed, pooled and centrifuged. The resulting supernatant was decanted and resuspended in a phosphate buffer to produce a 0.75 ml sample for Xpert testing.

### Statistical analysis

Data were analyzed using STATA 11.0 (StataCorp, College Station, Texas). Bivariate analyses employed Wilcoxon sum rank, chi-square and Fisher's exact tests, as appropriate. Logistic regression models were developed to examine the association between both LAM and Xpert^® ^positivity and CD4 strata. Mixed effect, random intercept models were developed to examine changes in LAM OD over time. Mean changes in proportion of patients with positive LAM results and the significance of trends pre and post week 2 of TB treatment were assessed using linear regression models. An autoregressive model was used to account for the autocorrelation of the data, and the impact of time was examined through an interaction term. All statistical tests are 2-sided at α = 0.05.

## Results

### Study cohort

**Figure 1:**
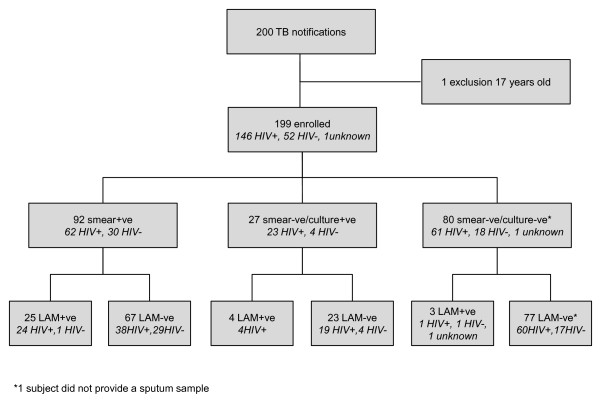
**LAM Study Consort Diagram**. Disposition by HIV and TB sputum bacteriological status of 200 consecutive patients presenting to a South African township TB clinic and undergoing LAM-ELISA urine testing

Two hundred consecutive adults were diagnosed and started on TB therapy in the clinic, and invited to participate in the study between September 2009 and April 2011. One subject was found to be under study entry age (17 years old) and the 199 met entry criteria and were recruited to the study. The TB diagnostic grouping, HIV status and urine LAM testing results are shown in Figure 1. 92/199 (46.2%) of patients were sputum smear positive and of the total sputum smear positive patients, 85/92 (92.4%) were culture positive and 7/92 (7.6%) were culture negative. 27/199 (13.6%) were sputum smear negative and sputum *Mtb *culture positive and 80/199 (40.2%) were presumptive TB cases (sputum smear and culture negative). HIV sero-status was positive in 146/199 (73.4%), negative in 52/199 (26.1%) and 1/199 (0.5%) refused HIV testing. LAM was positive in 32/199 (16.1%) of patients, and of those, 29/119 (24.4%) had laboratory confirmed TB, 25/92 (27.2%) were sputum smear positive and 24/62 (38.7%) were HIV positive smear positive.

The overall sensitivity of the LAM-ELISA assay in sputum smear or culture confirmed cases was 24.4%. Of the 146 HIV positive patients, the sensitivity was 69.2% in those with a CD4 count < 50 cells/μl, 42.9% in those with a CD4 count 50-99 cells/μl, 32% in those with a CD4 count 100-199 cells/μl, and 15.2% in those with a CD4 count of ≥ 200 cells/μl.

### LAM positive cohort

32/199 (16.1%) of patients were classified as LAM positive; 27 patients tested positive on day 1 and a further 5 patients, who were LAM negative on day 1, tested LAM positive on multiple occasions during TB treatment. Of the latter group, 2 patients had 2 positive LAM results (median LAM-ELISA OD = 0.195 and 0.228), 1 had 3 positive LAM results (median LAM-ELISA OD = 0.114), 1 had 4 positive LAM results (median LAM-ELISA OD = 0.169) and 1 had 5 positive LAM results (median LAM-ELISA OD = 0.44). 15 patients with single LAM positive urines were not classified as LAM positive.

### LAM analysis during TB treatment

**Figure 2:**
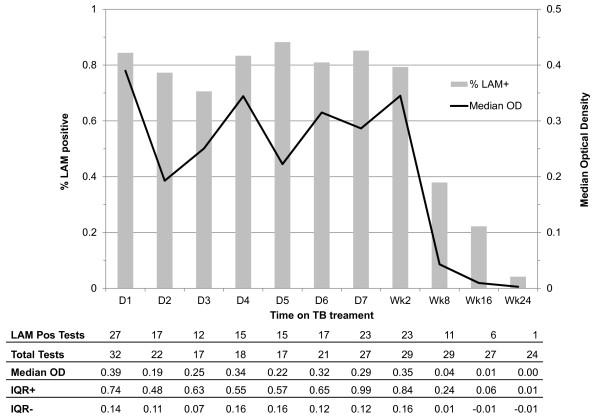
**Proportion of LAM positive patients testing LAM positive at days 1-7, week 2, week 8, week 16 and week 24 and median LAM-ELISA optical density at each time point**. Proportions are represented as bars (values shown on left-hand vertical axis) and median optical density is represented as a solid line (values shown on right-hand vertical axis). The number of patients who tested at each time point, median OD and IQR are shown in corresponding table below the figure

The number of urine samples collected from LAM positive patients, the proportion of samples testing positive and the median LAM-ELISA OD value at each time point during TB treatment are shown in Figure 2. OD measures decreased over the period of observation with a 0.004 unit reduction in OD per day (p < 0.001). However, OD measures remained stable during the first 2 weeks (p = 0.93), dropped significantly between week 2 and week 8 (p = 0.005), and although further decline continued from week 8 to week 24, this did not reach statistical significance (p = 0.36). Similarly, autoregression models showed that the proportion of all LAM positive patients who were LAM positive at each time point, remained stable in the first 2 weeks (p = 0.99), and decreased from week 8 to week 24, although this trend did not reach statistical significance (p = 0.77).

### LAM and host risk factors

Host factors including age, gender, patient category, site of TB disease, HIV status, CD4 cell count and antiretroviral status at baseline are presented for LAM positive and LAM negative patients in Table 1. Being LAM positive was not statistically related to age, gender, patient category, or routine designation of pulmonary and extra-pulmonary TB. However, LAM positivity was associated with laboratory confirmation of TB (*p *< 0.001) and being HIV positive (*p *= 0.002). Amongst HIV positive patients, LAM positivity was associated with lower median CD4 cell count (p = 0.006) but not with antiretroviral use (*p *= 0.078).

**Table 1:** Host factors and urine LAM Status of 199 study participants

Host Factors	Total n = 199	LAM Negative n = 167 (83.9%)	LAM Positive* n = 32 (16.1%)	p-value**
**Median age years [IQR]**	34 [28-43]	35 [30-40]	34 [28-44]	0.929†

**Gender**				0.904††

Male	110	92 (55.1)	18 (56.3)	

Female	89	75 (44.9)	14 (43.8)	

**Patient TB category**				0.968††

New TB case	125	105 (62.9)	20 (62.5)	

Re-treatment TB case	74	62 37.1)	12 (37.5)	

**Site of disease**				0.613‡

Pulmonary	168	141 (84.4)	27 (84.4)	

Extra-Pulmonary	18	16 (9.6)	2 (6.3)	

Pulmonary and Extra-Pulmonary	13	10 (6)	3 (9.4)	

**Smear or culture positive**				< 0.001‡

Positive	119	90 (53.9)	29 (90.6)	

Negative	79	76 (45.5)	3 (9.4)	

Not done	1	1 (0.6)	0 (0)	

**HIV status**				0.002‡

Positive	146	117 (70.1)	29 (90.6)	

Negative	52	50 (29.9)	2 (6.3)	

Unknown	1	0 (0)	1 (3.1)	

**Median CD4 cells/μL. [IQR]^a^**	132.5 [70-283]	156 [80-309]	95 [42-134]	0.006†

< 50	26	17 (14.5)	9 (31)	

50-99	28	22 (18.8)	6 (20.7)	

100-199	39	31 (26.5)	8 (27.6)	

≥ 200	53	47 (40.2)	6 (20.7)	

**ART at start of TB treatment^a^**				0.078††

Yes	33	30 (25.6)	3 (10.3)	

No	113	87 (74.4)	26 (89.7)	

*LAM positive patients are defined as those who were LAM positive on day 1 or on two or more occasions during TB treatment. A LAM result was considered positive when the average sample LAM-ELISA optical density (OD) minus the negative control was greater than or equal to 0.1 and negative when the average sample OD minus the negative control was less than 0.1. **p-values indicate differences between LAM positive and LAM negative participants. *p*-values were calculated using Wilcoxon rank sum^†^, chi-square^††^, and Fisher's exact test^‡ ^. ^a^of the 146 HIV positive subjects

**Figure 3:**
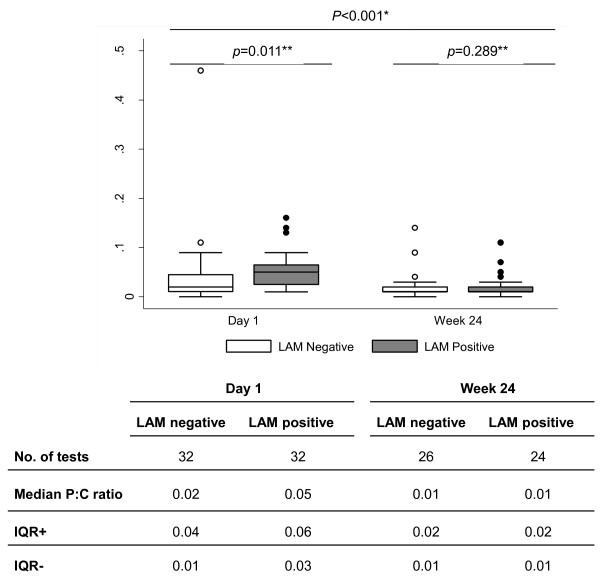
**Protein: Creatinine Ratios of 32 LAM positive patients and 32 randomly chosen LAM negative patients at Day 1 and Week 24 of TB treatment**. There was an overall statistically significant difference in P:C ratios between day 1 and week 24 (p < 0.001, Wilcoxon signed rank test*). There was a statistically significant difference in P:C ratios between LAM positive and LAM negative patients at day 1 (p = 0.011, Wilcoxon rank sum test **) but not at week 24 (p = 0.289, Wilcoxon rank sum test **)

The median values with interquartile ranges (IQR) of P:C ratios of 32 LAM positive and 32 LAM negative patients at day 1 and 24 LAM positive and 26 LAM negative patients at week 24 are shown in Figure 3. P:C ratios were higher on day 1 for LAM positive patients compared with LAM negative patients (p < 0.001) but similar for both groups at week 24 (p = 0.8). Elevated proteinuria (> 0.150 g/24 hours) was noted in 47 patients (28 LAM positive, 19 LAM negative) on day 1 and 15 patients (8 LAM positive, 7 LAM negative) at week 24. Moderate proteinuria (> 1 g/24 hours) was present in 4 patients (3 LAM positive, 1 LAM negative) at day 1 and 1 patient (LAM negative) at week 24.

Furthermore, 33/167 (19.8%) of LAM negative patients and 12/32 (37.5%) of LAM positive patients had a smear positive grading of +++, 9/167 (5.4%) and 5/32 (15.6%) were smear positive ++, 15/167 (9%) and 6/32 (18.8%) were smear positive +, 7/167 (4.2%) and 1/32 (3.1%) were smear positive scanty, and 103/167 (61.68%) and 8/32 (25%) were smear negative. Overall, LAM positive patients had a higher smear grading compared to LAM negative patients (p = 0.001).

### LAM and TB treatment outcomes

Treatment outcomes were similar in LAM positive and LAM negative patients. Deaths 2/32 (6.3%) and 8/167 (4.8%), treatment completion or cure 24/32 (75%) and 130/167 (77.8%), default/failure/transfer out 4/32 (12.5%) and 21/167 (12.6%), and miscellaneous 2/32 (6.3%) and 8/167 (4.8%) occurred in the LAM positive and LAM negative patients respectively.

### RFLP analysis

65 *Mtb *isolates were successfully cultured and underwent RFLP analysis, identifying 42/65 (64.6%) different unique strain codes. There was considerable genetic diversity between strains with 6/9 (66.6%) discrete synonymous single-nucleotide polymorphism-based (sSNP) phylogenetic lineages represented [37]. Identified strains were classified as belonging to CC 14/42 (33.3%), W-Beijing 6/42 (14.3%) and 22/42 (52.4%) a variety of other genotype families. 6/42 (14.3%) of strains were common to both LAM positive and LAM negative patients, 13/42 (31%) of unique strains were from LAM positive patients and 23/42 (54.8%) of unique strains were from LAM negative patients.

21/42 (50%) of strains had been previously recognised as circulating in this community between 2001 and 2006 [35] or identified in a more recent survey [36], while 12/42 (28.6%) of LAM positive and 9/42 (21.4%) of LAM negative strains were identified for the first time. We therefore found no evidence of LAM association with specific TB strains, TB strain families or sSNP phylogenetic lineages.

### Urine xpert^® ^MTB/RIF assay

**Figure 4:**
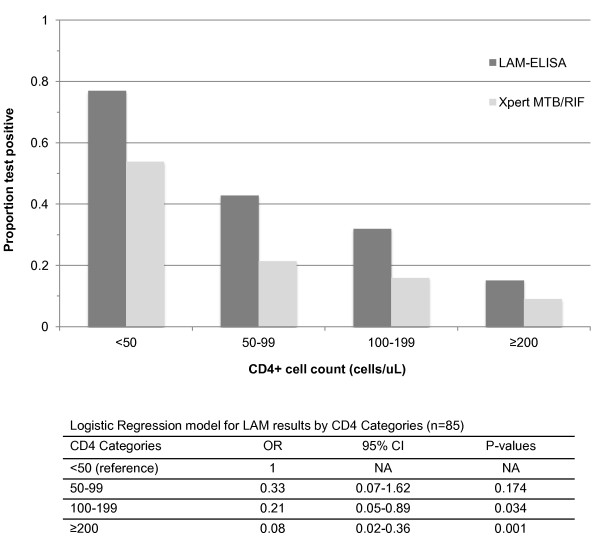
**Proportions of urine samples from 85 HIV positive patients with laboratory confirmed TB who were LAM positive and Xpert^® ^MTB/RIF positive stratified into 4 CD4 cell strata: < 50 cells/μl, 50-99 cells/μl, 100-199 cells/μl, and ≥ 200 cells/μL**. The proportions of LAM positive urine samples were 69.2%, 42.9%, 32%, 15.2% and Xpert^®^

10/32 (31.3%) day-1 urine samples and a further 5/22 (22.7%) of pooled urine samples tested Xpert^® ^MTB/RIF assay positive. All 15/15 (100%) Xpert^® ^positive urines were from LAM positive patients and none from LAM negative patients. Therefore all Xpert^® ^positive results occurred only in LAM positive patients. Similarly, there was a strong association between HIV and TB status; 14/15 (93.3%) Xpert^® ^positive tests occurred in those that were HIV positive with laboratory proven TB. One Xpert^® ^positive patient was HIV negative with presumptive TB. Of the 85 HIV positive patients with laboratory proven TB patients, the proportion that were LAM positive and the proportion that were Xpert^® ^positive are shown stratified by CD4 cell count in Figure 4. The proportions LAM positive were 9/13 (69.2%), 6/14 (42.9%), 8/25 (32%), 5/33 (15.2%) and Xpert^® ^positive were 7/13 (53.8%), 3/14 (21.4%), 2/25 (8%), 2/33 (6.1%), in CD4 cell count strata < 50 cells/μl, 50-99 cells/μl, 100- 199 cells/μl, and ≥ 200 cells/μL respectively. The bivariate logistic regression modelling showed that CD4 cell count strata are a significant predictor of LAM status with an Odds Ratio (OR) of 0.45 (95% CI: 0.28-0.72; p = 0.001). CD4 cell count was then stratified into 4 strata and a multivariate logistic regression analysis was done. With each increase in CD4 cell count strata, the OR of being LAM positive decreased: 0.33 (95% CI:0.07-1.62; p = 0.174), 0.21 (95% CI: 0.05-0.89; p = 0.034) and 0.08 (95% CI:0.02-0.36; p = 0.001) in CD4 cell count strata, 50-99 cells/μl, 100-199 cells/μl, and ≥ 200 cells/μL compared to < 50 cells/μl stratum as reference stratum, respectively. A similar trend between CD4 cell count

strata and Xpert^® ^exists, however it did not reach statistical significance with an OR of 0.50 (95% CI: 0.23-1.06; p = 0.07).

positive urine samples were 53.8%, 21.4%, 8%, 6.1% in CD4 cell count strata < 50 cells/μl, 50-99 cells/μl, 100-199 cells/μl, and ≥ 200 cells/μL respectively. The logistic regression models showed that CD4 cell count strata is a significant predictor of LAM status (OR = 0.45; p = 0.001). With each increase in CD4 cell count strata, the odds ratio (OR) of being LAM positive decreased. A similar pattern was seen with Xpert^® ^but did not reach statistical significance (OR = 0.50; p = 0.07)

## Discussion

In an adult South African TB clinic population approximately one quarter of patients with laboratory proven TB were found to have detectable LAM in urine. We found increased sensitivity of the LAM test in HIV infected individuals with advanced immune suppression, a finding which is consistent with previous studies [10,17,18,21,22]. Mild to moderate proteinuria was increased in both LAM positive and LAM negative HIV positive patients early in TB therapy which diminished with TB treatment. Heavy proteinuria sufficient to be associated with leakage of large immunoglobulins and immune-complex LAM was not recorded among LAM positive patients [27]. The major new finding of our study was the identification of *Mtb *organisms in the urine of nearly half of patients who were urine LAM positive and in none of the LAM negative controls. The presence of urinary LAM was not limited to a single infecting *Mtb *strain but was associated with an equally wide variety of individual strain patterns and strain families as found in LAM negative cases. Therefore, our findings indicate that urine LAM may be more related to the site of *Mtb *infection rather than the specific infecting strain.

Our initial hypothesis that the quantity of urine LAM as reflected by OD would be increased during early TB treatment was not substantiated. However, sensitivity of LAM testing was increased by inclusion of patients whose urine test became positive during TB treatment. It seems probable that the increased yield of LAM positive tests was due to increased sampling frequency rather than to increased release of LAM from organisms during therapy. However, the almost total clearance of LAM from the urine after 24 weeks of TB treatment likely reflects a measure of therapeutic response.

The Xpert^® ^MTB/RIF assay isolates mycobacterial organisms from clinical samples within the test cartridge by filtration prior to ultrasonic lysis which releases *Mtb *DNA for subsequent amplification [38]. We therefore used the Xpert^® ^assay as a measure of the presence of whole *Mtb *organisms within urine but recognized that the assay does not necessarily indicate the presence of viable organisms [38]. We found that almost half of the LAM positive patients tested Xpert^® ^positive using relatively small volumes of urine and therefore the proportion of Xpert positive results may have been underestimated. Larger urine sample volumes might have increased yield and allowed for *Mtb *culture which could have elucidated *Mtb *viability.

**Figure 5:**
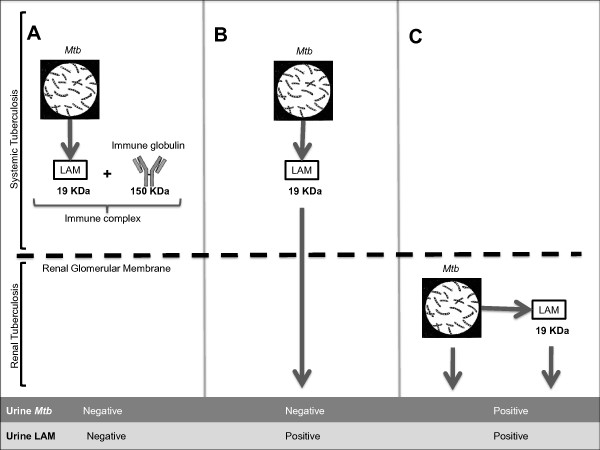
**Three proposed models of the possible fate of LAM molecules released from systemic or urinary *Mtb *organisms**. A. Systemically released LAM is bound to an antibody to form an immune complex within the circulation that impedes renal filtration of LAM across the intact glomerular membrane. This model gives rise to a negative LAM test in the absence of *Mtb*. B. Circulating LAM unattached to a specific anti-LAM antibody is freely filtered through the kidney into the urine, which gives rise to a positive LAM test in the absence of *Mtb*. C. *Mtb *within in the renal tract releases LAM directly into urine, which gives rise to a positive LAM test in the presence of *Mtb*.

Three proposed models of the possible fate of LAM molecules released from *Mtb *organisms in different body compartments are illustrated in Figure 5. Model 5A shows LAM released from organisms in the systemic (non-renal) compartment into the circulation where they bind to specific anti-LAM antibodies to form large immune complexes with limited capacity to pass through the normal kidney to the urine. In patients with normal renal filtration this model would be expected to give rise to a negative urine LAM test in the absence of mycobacteriuria. Model 5B reflects the currently accepted model of urinary LAM [14] in which free LAM molecules released from *Mtb *organisms in the systemic compartment into the circulation are not antibody bound and are therefore able to pass freely through the normal kidney into the urine. This model gives rise to a positive urine LAM test in the absence of *Mtb *organisms. Model 5 C is compatible with the findings of this study in which urine LAM is released directly from *Mtb *organisms within the renal tract compartment, which gives rise to a positive LAM test in the presence of *Mtb *organisms in urine. Mycobacteriuria presents evidence of extra-pulmonary TB with renal tract involvement. To determine the relative contribution of model 5 C to urine LAM, further studies will be required in which larger quantities of urine are processed to increase sampling probabilities.

We were not able to show whether detected urinary LAM existed as free LAM molecules, was complexed with anti-LAM antibodies, or was physically associated with mycobacterial organisms. However, a strong association between urine LAM detection and mycobacteremia has been previously reported [18] which may indicate a likely source for mycobacteriuria.

Although LAM ELISA had moderate sensitivity in our study overall, it is important to highlight the increased sensitivity and utility of this diagnostic amongst those with low CD4 count [39]. Screening for TB among patients with advanced immunodeficiency prior to antiretroviral therapy is challenging and rapidity is critical in view of high mortality risk [39]. However, a simple, low-cost, point-of-care version of the assay for detection of LAM (Determine TB-LAM) has now been shown to have considerable utility as a screening tool in this setting [22].

## Conclusions

In summary, this study determined factors potentially impacting the sensitivity of urine LAM for TB diagnosis in a South African population routinely attending a TB clinic. Sensitivity of LAM testing was independent of *Mtb *strain but was associated with advanced HIV infection and laboratory confirmed TB disease. However, the finding of urine LAM did not appear to indicate worse TB treatment outcomes. Qualitative and quantitative estimations of urine LAM may have future utility as biomarkers reflecting response to TB treatment. The isolation of *Mtb *from a large proportion of LAM positive cases indicates that renal TB may occur more commonly in advanced HIV than previously recognised. The LAM assay is a promising diagnostic for use in HIV positive patients with low CD4 counts and a new point-of-care version of this assay now enables rapid TB diagnosis in this patient population.

## Competing interests

No interpretations of data or presentation of information of this study was influenced by any of the author's personal or financial relationship with other people or organizations. None of the authors have any financial or non-financial competing interests.

## Authors' contributions

All authors made substantive intellectual contributions to this study. RW, KM, LGB, SDL contributed significantly to the study conception and design. RW, KR, KM, SDL contributed significantly to the data analysis and interpretation. KR supervised data collection and management. MV supervised specimen collected, analysis, and management. BNK supervised RFLP testing. All authors contributed significantly to the manuscript draft.

## Pre-publication history

The pre-publication history for this paper can be accessed here:

http://www.biomedcentral.com/1471-2334/12/47/prepub
